# Gender Influences the Initial Impact of Subarachnoid Hemorrhage: An Experimental Investigation

**DOI:** 10.1371/journal.pone.0080101

**Published:** 2013-11-08

**Authors:** Victor Friedrich, Joshua B. Bederson, Fatima A. Sehba

**Affiliations:** 1 Department of Neurosurgery, Mount Sinai School of Medicine, New York, New York, United States of America; 2 Department of Neuroscience, Mount Sinai School of Medicine, New York, New York, United States of America; University of Pecs Medical School, Hungary

## Abstract

Aneurysmal subarachnoid hemorrhage (SAH) carries high early patient mortality. More women than men suffer from SAH and the average age of female SAH survivors is greater than that of male survivors; however, the overall mortality and neurological outcomes are not better in males despite their younger age. This pattern suggests the possibility of gender differences in the severity of initial impact and/or in subsequent pathophysiology. We explored gender differences in survival and pathophysiology following subarachnoid hemorrhage induced in age-matched male and female rats by endovascular puncture. Intracranial pressure (ICP), cerebral blood flow (CBF), blood pressure (BP) and cerebral perfusion pressure (CPP) were recorded at and after induction of SAH. Animals were sacrificed 3 hours after lesion and studied for subarachnoid hematoma size, vascular pathology (collagen and endothelium immunostaining), inflammation (platelet and neutrophil immunostaining), and cell death (TUNEL assay). In a second cohort, 24-hour survival was determined. Subarachnoid hematoma, post-hemorrhage ICP peak, BP elevation, reduction in CPP, intraluminal platelet aggregation and neutrophil accumulation, loss of vascular collagen, and neuronal and non-neuronal cell death were greater in male than in female rats. Hematoma size did not correlate with the number of apoptotic cells, platelet aggregates or neutrophil. The ICP peak correlated with hematoma size and with number of apoptotic cells but not with platelet aggregates and neutrophil number. This suggests that the intensity of ICP rise at SAH influences the severity of apoptosis but not of inflammation. Mortality was markedly greater in males than females. Our data demonstrate that in rats gender influences the initial impact of SAH causing greater bleed and early injury in males as compared to females.

## Introduction

Aneurysmal subarachnoid hemorrhage (SAH) accounts for 5% of all stroke cases [1]. The initial impact of the SAH highly correlates with the outcome; approximately 45% of patients die within 30 days of SAH; 12% within the first 24 hours and 33% within 48 hours [1]. There is overall agreement that early treatment is necessary to save lives after SAH [2], however, the mechanisms of brain injury during this early period remain poorly understood and few specific treatments for them exist.

Gender influences the risk of SAH, and age influences its outcome. Females harbor a greater number of intracranial aneurysms than males [3,4]. The average age of female SAH patients is 4-5 years greater than males but the outcome, studied as average length of hospital stay, case fatality rates, and neurological outcomes, is similar [3,5,6]. This discrepancy (similar outcome among patients of two different average ages), suggests that, age aside, outcome is in fact more favorable in women than in men. Published reports show that more men than women die within the first 24 hours after SAH [7–9]. Consequently, gender appears to have an influence in the initial impact and early phase of SAH. 

 The present study explored whether gender differences exist in initial impact and early injury after SAH in an experimental (rat) model. SAH was induced via endovascular arterial perforation in age-matched male and female rats and cross-gender differences in early physiological measures (intracranial pressure, mean arterial blood pressure, cerebral blood flow and cerebral perfusion pressure), size of subarachnoid hematoma, vascular histology, brain inflammation and cell death, and mortality were studied.

## Methods

All animal procedures and protocols used in this study were reviewed and approved by the Animal Care Committee of the Mount Sinai Medical Center.

### Surgical preparation, physiological monitoring and SAH production

SAH was induced in six-month-old male (538±15 gm) and female (342±9 gm) Wister rats using the endovascular suture model. Rats were anesthetized with ketamine-xylazine (50mg/Kg+5mg/Kg IP), placed on a homoeothermic blanket (Harvard Apparatus) linked to a rectal temperature probe set to maintain body temperature at 37°C. Rats were further transorally intubated, ventilated, and maintained on inspired isoflurane (1-2% in 21% oxygen-supplemented room air). The right external carotid artery (ECA) was identified and exposed to its origin at the common carotid artery bifurcation. After distal ligation of the ECA, a temporary aneurysm clip was placed at the origin of the ECA while ensuring patency of the internal carotid artery (ICA). A 3’0 proline suture was advanced retrogradely through the ligated right ECA, and distally through the internal carotid artery (ICA) until the suture perforated the intracranial bifurcation of the ICA. SAH was confirmed by a rise in intracranial pressure (ICP) and a reduction in cerebral blood flow (CBF). The filament was then withdrawn into the ECA, reperfusing the ICA.

Each animal was returned to its cage as it regained consciousness and was able to breathe spontaneously. Animals were sacrificed 3 or 24 hours after hemorrhage (N=5 per time and gender).

Age- and gender-matched sham-operated animals (N=5 per gender and time of sacrifice) were used as controls in this study. As described previously, sham surgery included all steps carried out for SAH induction, except that perforation of the internal carotid artery was not effected [10].

### Blood Gas analysis

Femoral artery blood gases (pCO_2_, pO_2_; ABL, Radiometer America) and blood pH were measured prior to SAH induction. Blood gases were adjusted as required to ensure that they are normal prior to SAH induction. 

### Physiological measurements

Intracranial pressure (ICP) and systemic blood pressure (BP) were monitored using pressure transducers (PT300, Grass Instruments, USA). Cerebral blood flow (CBF) was monitored using Laser Doppler Flowmetry (LDF; Vasamedics Inc. USA). Physiological measurements were recorded in real time using PolyView software (Grass Instruments, USA). Recording began 20 minutes before induction of SAH and lasted to at least 60 minutes after induction of SAH. Cerebral perfusion pressure (CPP) was calculated (CPP = BP-ICP). We used peak ICP and 60-minute CBF values to monitor the intensity of SAH in each rat. 

#### Mean arterial blood pressure (BP)

The right femoral artery was exposed and cannulated for blood gas and blood pressure monitoring. 

#### Intracranial pressure (ICP)

The occipital muscles were dissected from the occipital bone and retracted caudally. A 25-gauge needle was used to puncture the atlanto-occipital membrane and a PE-50 catheter (Intermedics Inc) was inserted under direct visualization into the cisterna magna for continuous ICP measurement. Acrylic cement was used to secure the ICP catheter to a stainless steel screw anchored in the occipital bone. 

#### Cerebral blood flow (CBF)

Skin over the coronal suture was removed and the underlying bone was thinned. A Laser Doppler Flowmetry (LDF) probe (0.8mm diameter, model P-433, Vasamedics Inc.) was advanced with stereotaxic guidance and placed at a location immediately adjacent to the coronal suture and 5 mm lateral to the right of midline, over the territory of the middle cerebral artery and away from large meningeal vessels. 

#### Post surgery monitoring and treatment

At the completion of surgery animals were returned to single warm cages. Animals were monitored for pain, seizures, and weight loss. For pain management animals were injected with Buprenorphine 0.05mg/kg, SQ, every 12 hours. For weight management animals were injected with Ringer lactate (1ml, IP) for fluid replacement. In addition, animals were allowed free access to water and special diet (gel) and wet pellets (are easier to chew), to prevent weight loss. 

In our animal protocol an animal is euthanized if it experiences seizures, excessive weight loss or exhibits signs of pain.  None of the animals used in the present study required euthanasia.

#### Sacrifice

For sacrifice each animal was anesthetized (Ketamine-Xylazine; 50mg/5mg/Kg; IP) and then subjected to gentle gravity-driven intra cardiac perfusion of chilled saline (250 ml). The brain was removed immediately after perfusion and frozen. Previously we have found that this method removes the blood from the vessels but preserves vascular platelet aggregates and adherent neutrophils in place [10–12] . 

### Histology

We assessed inflammation and apoptosis at 3 hours after SAH, as our previous studies showed that both phenomena are robust at this time [10,12] . Coronal sections at bregma -3.8, - 0.12, and +1.2 [13] were prepared by cryostat (8 μm thickness) and stored at -70 ° C. 

### Measurement of subarachnoid hematoma size

Hematoma size was measured in sections from animals sacrificed 3 hours after SAH surgery. Briefly, images of whole coronal sections were acquired and the total area of brain and the areas of brain-adherent blood in the basal subarachnoid space, cortical convexities, and interhemispheric fissure were determined by manual tracing [14]. The severity of hemorrhage was determined for each animal as the ratio of summed area of blood to area of brain. In our experience, in perfusion-fixed specimens much subarachnoid blood is fixed to the brain surface during perfusion and adheres to the brain surface when the fixed brain is removed from the cranium. We recognize that hematoma might in some cases be partially adhered to the cranium or be otherwise lost during the histological preparation; for that reason, values obtained for some individuals might be lower then the true intracranial hematoma size. Nevertheless in our experience the area of adherent blood as measured in sections is a reasonably good indicator of the original size of hemorrhage [14].

### Immunofluorescence

Primary antibodies were: goat anti-collagen-IV (1340-01, Southern Biotech. Inc.), FITC-conjugated rabbit anti-thrombocyte (FAD51440, InterCell Tech.), mouse anti-rat endothelial cell antigen (RECA-1; MCA970R , Serotec), mouse anti-NeuN (MAB377, Chemicon International), and rabbit anti-neutrophil (HB-199, the generous gift of Dr. D. Anthony, Oxford England). Secondary antibodies were species-specific donkey anti-goat IgG-AlexaFluor 647 and -AlexaFluor 350 (Invitrogen Corp. USA), donkey anti-mouse IgG-AlexaFluor 488 (Invitrogen Corp. USA), and donkey anti-rabbit IgG-Rhodamine Red-X (Jackson ImmunoResearch, USA).

 Primary antibodies, distinguished by species, were mixed and applied together with overnight incubation. After washing, species-specific secondary antibodies (all raised in donkey) were applied together and incubated overnight. Primary antibody combinations included collagen-IV and RECA-1 with or without either anti-thrombocyte or HB-199. 

### Vascular Constriction

The internal circumference of collagen-IV-stained major cerebral vessels of circle of Willis, the internal carotid artery and the A1 segment of proximal anterior cerebral artery, were measured in sections as described previously [15].

### Cell death

#### Apoptosis

Caspase-3 activity was assessed using APO LOGIX™ kit (SR-DEVD-FMK; Cell Technology Inc, CA, USA). Briefly, brain sections were thawed and incubated with fluoromethyl ketone (FMK) caspase inhibitor for 60 minutes at 37°C. The sections were then washed and immunostained for collagen IV and NeuN as described previously [16]. 

Apoptosis was assessed using the In Situ Cell Death Detection Kit (POD, Roche Applied Science, USA). Briefly, sections were incubated in permeabilization solution for 10 minutes and then incubated in TUNEL reaction mixture at 37°C for one hour. Brain sections were washed and immunostained for NeuN and collagen-IV as described previously [16]. 

Sections were also examined using Fluoro-Jade B staining. Air-dried sections were incubated in 100% ethanol for 3 minutes and 70% ethanol for 1 minute and then washed with deionized water. Sections were then incubated for 10 minutes in 0.06% potassium permanganate followed by 30 minutes in Fluoro-Jade B solution (0.001%. Histo-Chem, Inc., USA) with added DAPI. Sections were dried overnight at room temperature, cleared with xylene, and coverslipped using DPX (Electron Microscopy Sciences Inc.USA). 

### Morphometry

20x widefield (image field area= 8 x10^4^ μm^2^, Zeiss Axioplan 2) and confocal (Leica TCS-SP) images were collected at standard locations in olfactory tubercle, cerebral cortex, hippocampal formation, striatum, and lateral and dorsal cerebral cortex. Locations were: in olfactory tubercle, two fields medial and lateral within the region; in caudoputamen, three regions located at dorsomedial, lateral, and ventral positions; in hippocampus, CA1 and dentate gyrus. The dorsal cerebral cortex sample included M1, M2 and dorsal S1 and the lateral cerebral cortex sample included lateral S1, S2 and insular cortex. Constant illumination and exposure settings were used for images collected for quantitative analysis.

Quantitative studies were performed by an observer blinded to specimen identity. 2-3 fields per region per hemisphere per animal were analyzed using IPLab software (Scanalytic Inc, vr 3.63; USA). The aggregate area fractions of RECA-1 and collagen IV positive profiles and the number of vascular luminal platelet aggregates and of neutrophils were determined as described previously [11,12]. Numbers of neurons and of endothelial cells positive for Caspase-3 and TUNEL and the number of Fluoro-Jade B-positive cells were obtained manually [16].

### Statistics

Data were analyzed by two-way ANOVA with interactions (Stat View v 5.0.1, SAS institute Inc. USA) followed, where appropriate, by Fisher’s PLSD post-hoc t-tests. Regression analysis to assess correlations between ICP peak or subarachnoid hematoma size and the numbers of vascular platelet aggregates, neutrophils, and apoptotic cells was also performed (Stat View v 5.0.1, SAS institute Inc. USA). Survival analysis: At 24 hours after SAH, five males (100%) were dead and none were alive; no females were dead and five (100%) were alive. These counts were analyzed using 2x2 (alive-dead x male-female) chi-square analysis with Yates' correction [17].

## Results

### SAH Physiological Parameters

ICP and CBF varied significantly and substantially with time in patterns well documented in previous studies [1]. 

#### ICP

**Figure 1:**
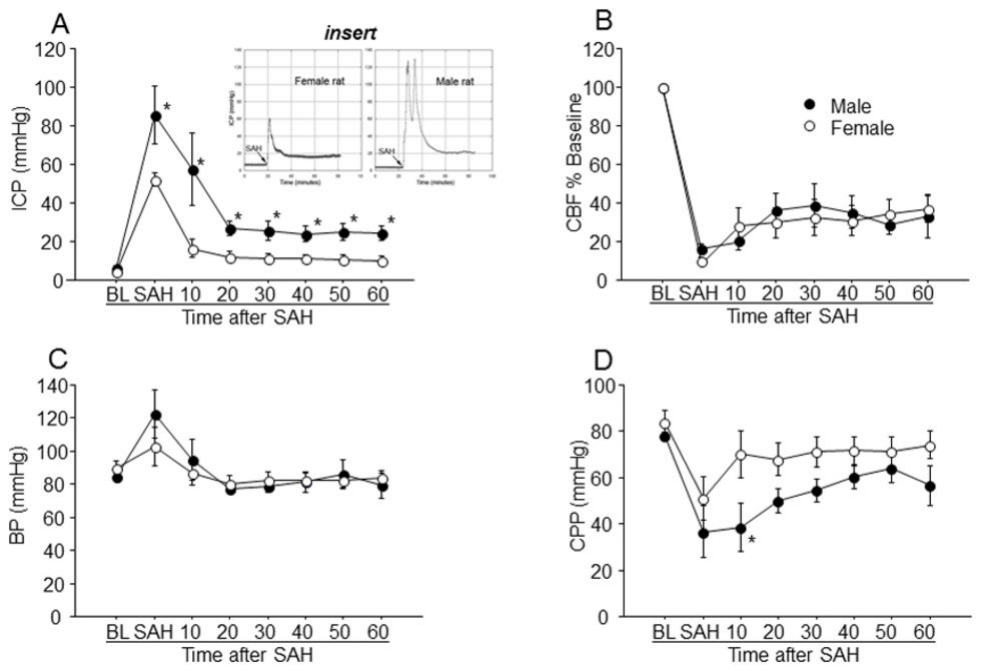
Gender differences in early physiology of SAH. ICP, CBF, and BP were measured in the real time from 20 minutes before to 60 minutes after SAH induction. CPP was calculated (BP-ICP). In panel A note that ICP peaked and declined to greater values in males than in females after SAH. Inserts in panel A shows ICP recordings from a single male and a single female rat; note multiple ICP peaks in the male and not in female. Panels B and C show similar cross-gender differences in CBF and BP post SAH. In panel D note different profiles of CPP changes in males and females after SAH. Data are mean ± sem from 10 animals per gender. * significantly difference from females, p <0.01).

ANOVA indicated a significant effect of gender (F=17.1; p<0.05; Figure 1A). Pre-hemorrhage ICP (baseline - BL, Figure 1A) was similar in males and females (males: 6.1 ± 1.1; females: 4.1 ±0.6 mm Hg); however, the maximum ICP at SAH was markedly greater in males than in females (males: mean: 86 ± 15.0 mmHg; females: mean: 52 ± 3 mmHg; p<0.05). After peaking, the ICP fell in both males and females; however, the 60-minute ICP in males (24.5 ±3.6 mm Hg) was more that twice that in females (9.8 ±2.6 mm Hg), a substantial and statistically significant difference (p<0.05; Figure 1A). In human adults an increase in ICP above 20 mm Hg is considered abnormal and above 25 mmHg requires aggressive treatment [18].

#### Multiple ICP peaks

After the elevation and decline of ICP following initial hemorrhage, subsequent re-elevations in ICP are seen when arteries rebleed [19]. We observed more then one ICP peak in 30% of females and 90% of males. The value of the second ICP peak was greater than first ICP peak in one female and in six male rats. The time between the two peaks was less than 5 minutes in females and ranged from less than 1 minute to 11 minutes in males (Figure 1A, insert).

#### CBF

No effect of gender on the extent of fall and the extent of recovery of CBF was found during the first 60 minutes after SAH. (F=0.04, p>0.05; Figure 1B). In males CBF fell to 16± 3.0% of baseline at SAH and recovered to 33.0 ± 11% after 60 minutes. In females CBF fell to 11 ± 2% of baseline at SAH and recovered to 37 ± 7% after 60 minutes.

#### BP

Baseline systemic blood pressure was similar in males and females (males: 85 ± 2; females: 90 ± 5 mmHg). Systemic blood pressure rose significantly in males, to 131% of baseline immediately after hemorrhage (effect of time: F= 3.1; p<0.05; Figure 1-C). By contrast, BP in females showed changes which were not statistically significant (effect of time: F= 1.4; p>0.05; Figure 1-C). After the first ten minutes post-hemorrhage, BP in both genders returned to near baseline values (Figure 1C). 

#### CPP

Baseline ICP was similar in males and females (males. 78 ± 1.2; females, 84.0 ± 5.3 mmHg). At SAH, CPP fell dramatically in both genders (males: 37 ± 11 mmHg, females 51 ± 9 mmHg; p<0.05) and then recovered during the succeeding 60 minutes to levels lower than baseline. ANOVA indicated a significant effect of gender (F=17.8; p<0.05); as is evident in Figure 1D, CPP in females recovered more rapidly and to levels nearer baseline than did CPP in males. CPP in females remained significantly greater than males at all times from 10 min after SAH through the end of the monitoring period (F=15.7, p<0.05).

### Subarachnoid Blood

Following hemorrhage, blood pooled in the subarachnoid space and surrounded the major cerebral arteries. A thin layer of blood extended across the hemispheres. As expected, we found an effect of gender on the area of brain in our coronal sections and on the total blood volume; male brains were 6.4% bigger in sectioned area (p = 0.07; data not shown). Hematoma size normalized for brain size (ratio bleed area to brain) was not significantly different between males and females (Figure- 2; p>0.05). Males exhibited notably greater variability than females in the total size of hematoma, perhaps the result of more frequent and variable rebleeds in males than in females. Hematoma size in individual animals ranged from 0.2 to 11 % of brain area in males and 0.3 to 6 % in females. 

**Figure 2:**
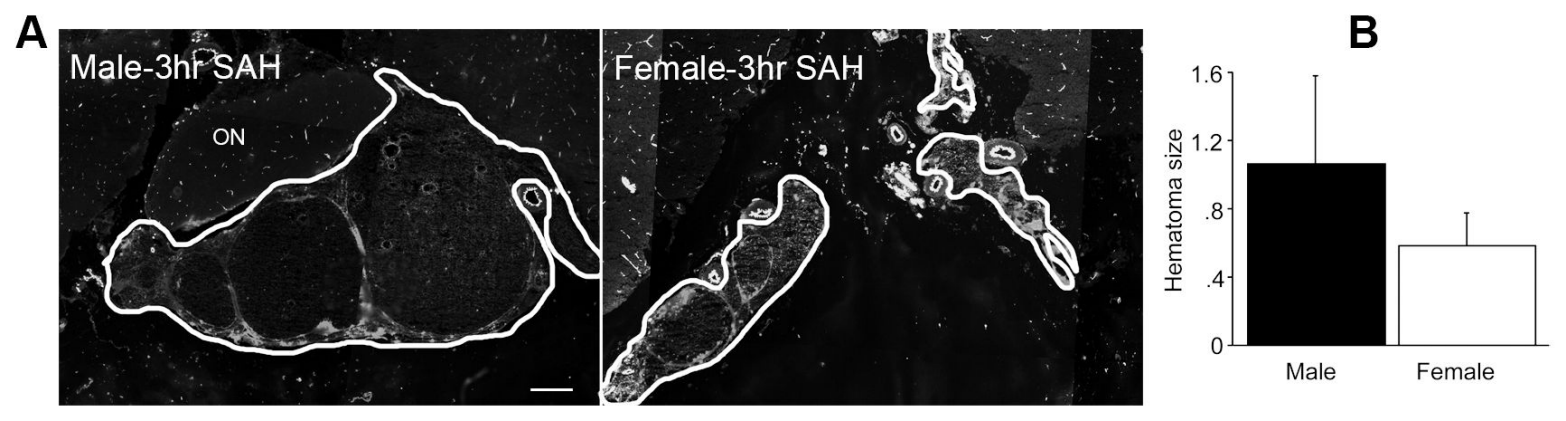
Subarachnoid hematoma. Panel A: representative images of subarachnoid blood in male and female rats at 3 hours after SAH. Subarachnoid clots (outlined in white in the images) exhibit a characteristic granular immunofluorescence for RECA-1 and are both surrounded by and infiltrated by neutrophils. In general, individual clots in males were larger and clots in females were smaller. ON: optic nerve; scale bar = 200 μm Panel B: the summed areas of subarachnoid blood were determined by tracing. The accumulated data show areas larger in males than in females, a trend which did not reach significance (p=0.4). Data are mean ± sem from 5 animals per gender. Cryostat sections from animals sacrificed at 3 hours after SAH and immunofluorescent for RECA-1 and neutrophils (HB-199) were used for this determination. As stated in Methods, the perfusion fixation procedure employed caused subarachnoid blood to adhere to the brain surface during removal of brains from the cranium. The grayscale images combine signals from two color channels.

### Constriction of Major Vessels

**Figure 3:**
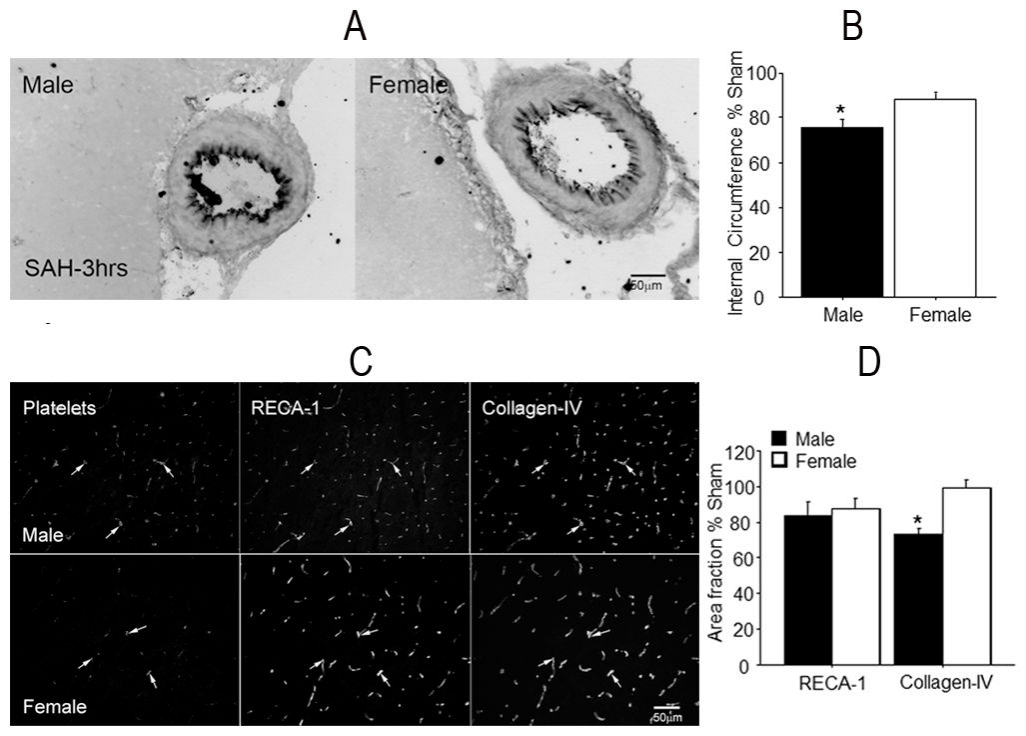
Vascular pathology. Cerebral vessels of animals sacrificed 3 hours after SAH. Panel A: representative images of ICA from a single male and a single female rat. Panel B: average vessel sizes. Note that the internal circumference of ICA in SAH males is smaller compared to females. Panel C: representative images showing brain vessels stained for platelets, RECA-1 (an endothelium marker), and collagen-IV (a basal lamina marker); note the greater numbers of RECA-1 and collagen IV stained vessels containing platelet aggregates (arrows) in males. Panel D: average area fractions of RECA-1 and collagen-IV positive vascular profiles of SAH animals as percent changes over sham-operated cohorts. The reduction in the area fraction of RECA-1 is similar in males and females but that of collagen -IV is different. Data are mean ± sem from 5 animals per gender. * significantly difference than females (p <0.01).

Diameter changes in internal carotid artery (ICA) and proximal anterior cerebral artery (A1 segment) were similar; consequently, their data were pooled for analysis [15]. At 3 hours post SAH the internal circumference was reduced significantly in males, by 25% (p<0.05). In females a smaller reduction of 12% was of marginal significance (p>0.05; Figures 3A and B). A comparison of male to female SAH animals showed difference at p=0.059 (F=3.6).

### Parenchymal vessels

#### Endothelium and Basal lamina

We used two-color simultaneous immunofluorescence with RECA-1 antibody, for endothelium, and anti-collagen-IV antibody for basal lamina, to visualize vessel profiles in parenchyma. RECA-1 and collagen-IV immunostaining revealed large numbers of vascular profiles in sham-operated male and female rats, and noticeably fewer such profiles in SAH animals. Though reduced in comparison to the shams, stained profiles appeared to be greater in number in SAH females than SAH males (Figure 3C). For quantitative documentation of this phenomenon, we determined the area fractions of RECA-1 and collagen-IV positive profiles in sham and in SAH animals. The area fraction of collagen-IV positive profiles was nearly identical in male (0.020 ± 0.001) and female (0.023 ± 0.001) sham-operated animals. After SAH, collagen-IV positive profiles were reduced substantially in males (31 % of sham value; p<0.05) but remained virtually unchanged in females (Figure 3D). Area fractions for RECA-1 trended lower in both genders after SAH, but differences were not statistically significant (Figure- 3D).

### Inflammation

#### Neutrophil infiltration

**Figure 4:**
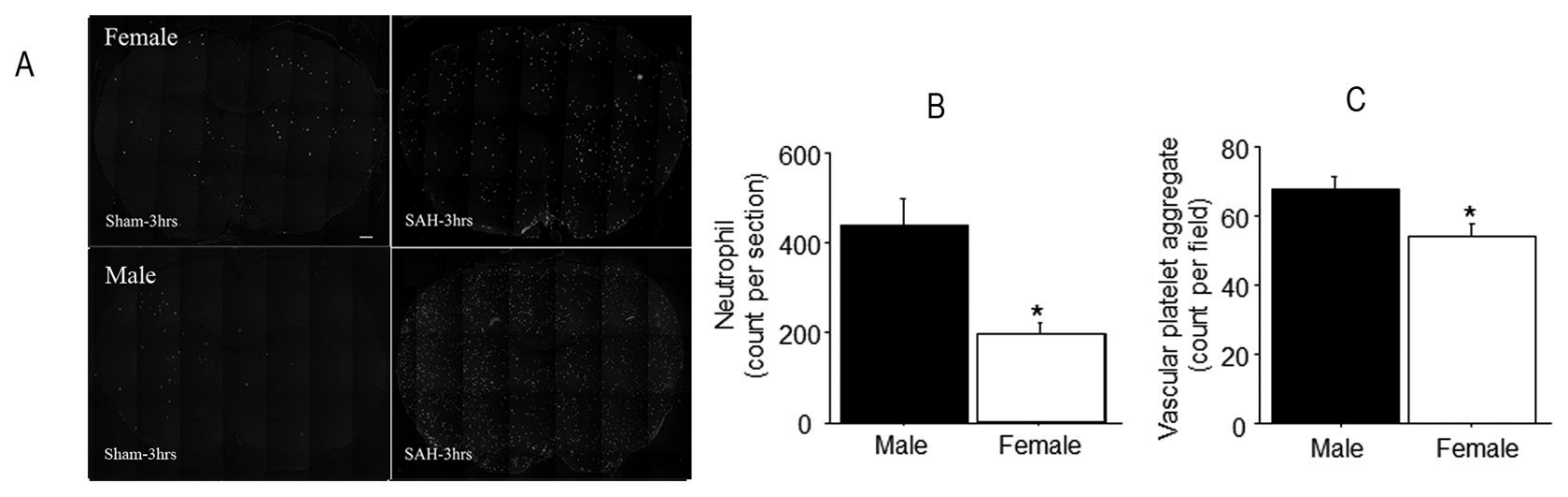
Cerebral inflammation. Luminal platelet aggregates and neutrophil accumulation in animals sacrificed 3 hours after SAH. Panel A: representative images of neutrophil staining. Note the greater number of neutrophils in male as compared to female brain. Scale bar = 500 μm. Panels B, C: average numbers of neutrophils and vascular platelet aggregates per whole brain section and per image field, respectively. Both parameters are greater in male as compared to female brains. Data are mean ± sem from 5 animals per gender. * significantly difference than females (p <0.05).

HB-199 was used to visualize neutrophils in the brain sections [12]. Neutrophils were seen at low numbers in sham operated animals of both genders (Males: 90 ± 16, females: 79 ±14 per brain section; Figure 4A). Following SAH, neutrophils were dramatically increased in number, with substantially more neutrophils in males (433 ± 59.7) than in females (193.7 ±29.5; p<0.05 for females vs males; Figure 4B). Close examination of enlarged images showed that in both genders about 57% of those cells had exited from the vascular lumen into the parenchyma (p>0.05) for gender effect). 

#### Platelet aggregation

Little platelet staining was evident in sham operated animals. By contrast, platelet staining was abundant after SAH (Figure 3A). We combined neutrophil and collagen-IV staining to determine the number of collagen stained microvessels profiles which contained platelet aggregates. In sham-operated animals only a few microvessels contained platelet aggregates, and their number was identical across genders (males: 10 ± 1; females 9 ± 1 per brain image; p>0.05 for gender effect). Aggregate-containing vessel profiles increased in number following SAH (Figure 4C), with a significantly greater increase in males (67 ± 4 per brain section) as compared to females (54 ± 4 per brain section; p<0.05 for gender difference).

### Cell death

Apoptosis was detected by caspase-3 activity and TUNEL assay.

#### Caspase-3 activity assay

**Figure 5:**
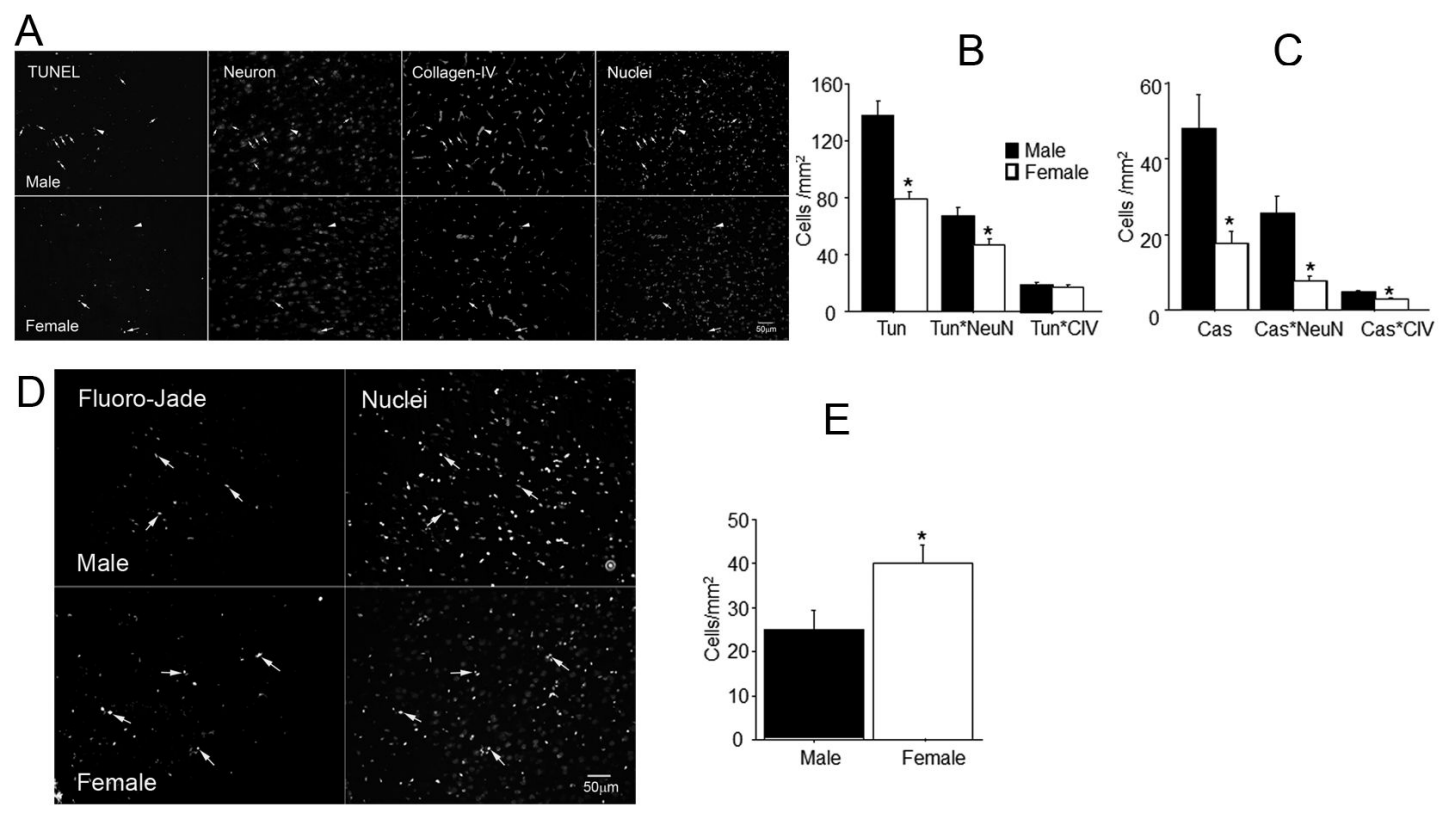
Cell death. Activated caspase-3 immunoreactivity and TUNEL staining 3h after SAH. Panels A-B: 4-color fluorescence staining for TUNEL, NeuN, collagen-IV, and DAPI. Panel A: typical micrographs from male and female SAH animals. Each channel is shown as a separate image. Small arrows: TUNEL-positive neurons; large arrowheads: TUNEL-positive vascular cells. Note the greater frequency of TUNEL-positive neurons in male as compared to female. Panel B: average numbers of TUNEL-only, TUNEL+NeuN, and TUNEL+collagen-IV profiles. TUNEL-only and TUNEL-NeuN profiles are significantly greater in males than in females. Panel C: average numbers of profiles positive for activated caspase-3 (Cas) only, Cas+NeuN, and Cas+collagen-IV. All three indexes are significantly greater in male animals. Panel D: Fluoro-Jade B-positive cells (arrows) in representative SAH male and female brain sections. Panel E: Average numbers of Fluoro-Jade B-positive cells in SAH animals. Data are mean ± sem from 5 animals per gender. * significantly gender difference (p <0.05).

Only a few cells contained Caspase-3 activity in sham-operated animals. By contrast, caspase-3 positive cells were distributed throughout the brains of SAH animals. The number of cells positive for caspase-3 activity was identical in sham operated males (6 ± 0.7 cells/ mm^2^) and females (6± 0.9 cells/ mm^2^). The number of caspase-3 positive cells increased after SAH and was significantly greater in males (48 ± 9/ mm^2^), than in females (18 ± 3/ mm^2^; p<0.05 for gender difference). Collagen IV and NeuN immunostaining of brains sections assayed for caspase -3 activity established that the majority of cells positive for caspase-3 activity were also positive for NeuN (Figure 5A) and were located in the parenchyma, while a smaller number of NeuN-negative cells were mainly associated with the vasculature.

#### TUNEL assay

TUNEL-positive cells were scattered throughout the brains of SAH animals. Many of these cells stained positive for NeuN and some for collagen IV (Figure 5B). The number of TUNEL-positive cells was significantly greater in SAH males than females (males: 137 ± 10; females: 77 ± 6 cells/mm^2^; p<0.05). We found no significant difference between hemispheres in the number of TUNEL-positive cells in either males or females. Sections triply stained for TUNEL, NeuN and collagen IV revealed that the majority of TUNEL-positive profiles were neurons, while a smaller number were non-neuronal and were associated with the vasculature (Figure 5C).

Fluoro-Jade B staining was also examined (Figure 5D) since in some instances it has been reported to indicate neurodegeneration [20,21]. Surprisingly, the number Fluoro-Jade B-positive cells was significantly greater in females than in males (females: 40±4, males: 25± 4 cells/mm^2^; p<0.05; Figure 5E). 

## Correlations

**Figure 6:**
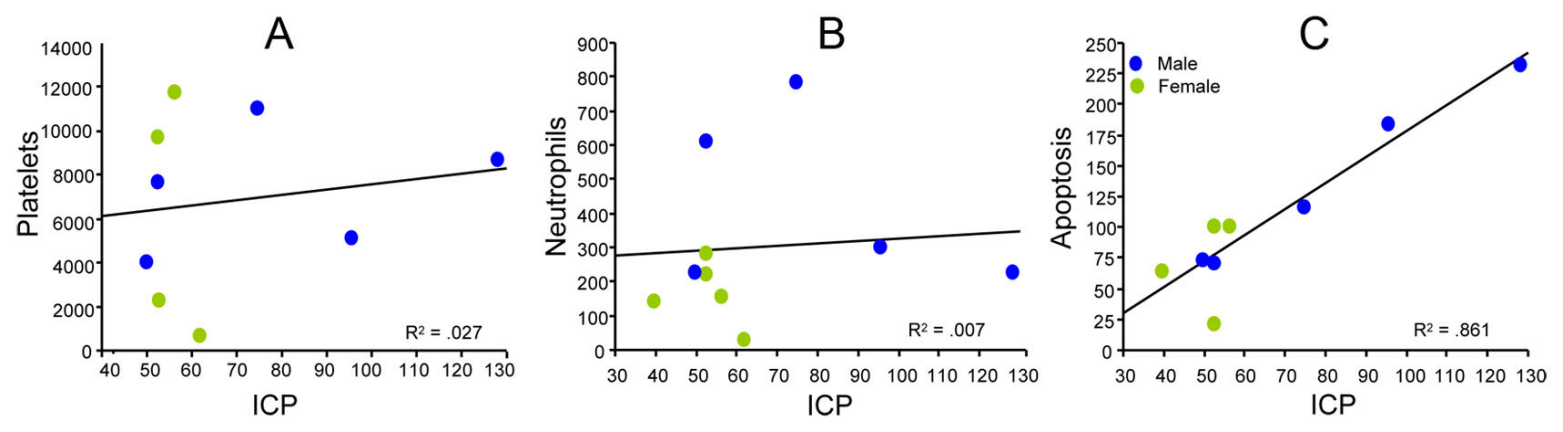
Correlation analysis. The association of ICP peak values with numbers of platelet aggregates (A), neutrophils (B), and apoptotic cells (C). The ICP peak significantly correlated with the number of apoptotic cells but not with the numbers of platelet aggregates or neutrophils. Each point is the mean from a single animal.

As expected, the ICP peak correlated significantly with subarachnoid hematoma size (p<0.05; data not shown), but no significant correlation was found between animal body weight and ICP peak or hematoma size (p>0.05). Peak ICP was a strong predictor of the number of apoptotic cells (p<0.05) but a poor predictor of the number of vascular platelet aggregates and neutrophils (p>0.05; Figure 6)

### 24 hour survival

All of the five females subjected to SAH and assigned to the 24 hour survival group did indeed survive to 24 hours post-SAH. None (0) of the five males assigned to the 24 hour post-SAH survival group survived for 24 hours. 24 hour survival in the sham operated group was 100% for both genders (Table 1).

**Table 1:** The number of animals surviving 24 hours post surgery.

**Gender**	**Sham**	**SAH**
**Male**	**100% (5/5**)	**0% (5/5**)*
**Female**	**100% (5/5**)	**100% (5/5**)

All sham-operated animals were alive at that interval; of SAH animals, all females were alive and all males had died. * mortality among male SAH animals was significantly greater than that among female SAH animals (chi-square (3) = 6.4; p<0.05).

## Discussion

We investigated the influence of gender on the initial impact of SAH in rat. We found that males experienced greater subarachnoid bleed, greater rise in intracranial pressure, greater vascular and brain injury, and greater 24 hour mortality than females. These results demonstrate a significant influence of gender in the initial impact of SAH, with severity of injury and outcome worse in males than in females. 

### Gender and early physiology of SAH

Elevation of ICP leading to excruciating headache is characteristic of SAH and is caused by intracranial volume loading and a concomitant increase in cerebrospinal fluid outflow resistance. In the present study, SAH elicited a significantly greater and more prolonged elevation in ICP and more rebleeds in males as compared to females.

Cross-gender differences in ICP elevation or in the size of subarachnoid hematoma have not been reported in human. However, at least one study notes a greater number of aneurysm rebleeds in men [22]. The authors speculate that this difference might reflect gender differences in the location of the aneurisms. In our study, all animals received the same procedure of SAH induction, perforation of ICA; rupture location was constant and can be ruled out as the source of the more frequent rebleeds in males. Gender-based difference in vascular biology or in clot formation could have contributed to our results. Differences in the rate of hemostasis between premenopausal women and men of a similar age are established [23,24].

The ICP decline which follows the initial ICP peak also displayed cross-gender difference in the present study. Whereas in females ICP declined to near baseline value, in males it remained significantly greater. Prolonged ICP elevation associates with poor patient prognosis and is a mass effect resulting from enlarging hematoma or acute hydrocephalus [25]. The prolonged ICP elevation observed in this study in males, which may have resulted from a greater volume of bleed or reduced CSF outflow, might contribute to the observed 100% 24 hour mortality.

We found a significant cross-gender difference in BP response to SAH; BP increased transiently in males but not in females. This difference may have resulted from the greater, near systolic pressure ICP elevation in males. Since brain perfusion is maintained by the difference between BP and ICP (CPP = BP-ICP), rises in ICP to or above the systolic pressure at SAH necessitates a reciprocal rise in BP to maintain the proper blood flow (the Cushing reflex [26]). The lower ICP elevation in females did not stimulate a BP rise. BP in both genders returned to near baseline values at ten minutes after SAH, the time when ICP and CPP in males remain greater than in females. This may indicate that the Cushing response is greatly diminished by 10 -15 minutes after initial bleed. Others report similar temporal recovery of BP after SAH [27].

In the present study the fall in CPP after SAH was greater and the recovery of CPP was less complete in males, which exhibit the Cushing response, compared to females, which do not exhibit the Cushing response. This finding is consistent with previous reports by others that the Cushing response does not always succeed in maintaining adequate cerebral perfusion and in fact can have deleterious rather than beneficial effects on the brain [28,29]. We did not observe hyperemia or CBF elevation in males or females after SAH. Quite remarkably, the initial fall in CBF as measured by Doppler flowmetry and its subsequent recovery were similar in males and females. In this regard, we note that, when ICP reduces CPP to below 50 mmHg (normal CPP in rat = 70 to 90 mmHg), microvascular flow is redistributed from capillaries (5-8 μm) to a large-diameter (8-15 μm) microvascular shunt system, a shift which is accompanied by brain hypoxia and edema and pathologically elevated CBF (hyperemia) [30]. This phenomenon has been observed in cerebral cortex, the area used for measuring CBF in our study. We believe that our Doppler flowmetric measurements, which report total flow, would not detect the presence of flow redistribution. Flow redistribution, if it occurred in our animals, could account both for gender-symmetry of the LDF measurements and for the greater early injury and mortality of the males. In SAH patients ICP rise coincides with the reduction in CBF. Perhaps measurements from deeper brain areas (such as basal ganglia and others) will provide a more complete picture of CBF changes after SAH. 

The LDF measurements in our studies are routinely used to register SAH (CBF falls upon artery perforation) and to predict 24 hour animal survival; animals with CBF greater than 40% of the baseline are more likely to survive the first 24 hr post SAH [31]. The 24 hr survival predicting power of 60 minute CBF however has been studied in male rats only. The results from the present study confirm that result for males but not for females; all females with CBF below 40% survived. This finding suggests that importance of CBF reduction should be reevaluated in context of survival and gender.

### Gender and early pathology of SAH

Previous studies show that cerebral vessels and neurons are affected very early after SAH [16,32]. More specifically, large vessels surrounding the circle of Willis constrict and the structure and function of parenchymal vessels are altered within minutes to hours after SAH. In male rats, constriction of large vessels occurs within 10 minutes and persists for at least 6 hours (for review see 32). The present study finds that although large cerebral vessels constrict in females, constriction is minimal. One factor that may have contributed to this cross-gender difference is the amount of blood pooled in the subarachnoid space upon SAH. This blood stretches the arachnoid membrane and this stress is mechanically transferred to associated vessels, eliciting vascular constriction[33]. Other factors that contribute to the acute constriction include sympathetic activation, reduction in nitric oxide levels, and increases in endothelin-1 after SAH (for review see 1). 

Parenchymal vessels endure severe structural damage and functional compromise after SAH. Structural damage is observed as loss of luminal surface antigen, detachment of endothelial lining from basement membrane, and degradation of collagen IV and laminin, which are the major proteins of the vascular basement membrane [32]. Functional compromise is observed as perfusions deficits and increases in permeability. The present finding that RECA-1 and collagen IV immunostaining of parenchymal vessels is reduced in SAH males is consistent with our previous findings [12,34]. This is the first study from our laboratory on the sequellae of SAH on female rats, and the observed smaller intensity of these changes in females is a new discovery. That female endothelium has greater resistant to ischemic injury is well established and is partially attributed to higher plasma levels of epoxyeicosatrienoic acids [35], potent vasodilators and suppressors post-ischemic inflammation. Their metabolic products are by contrast potent vasoconstrictors [36]. It is interesting to note that present study finds greater large vessel constriction and greater inflammatory response in male as compared to female rats after SAH.

Cerebral inflammation after SAH is observed as activation, intravascular accumulation, and parenchymal migration of leukocytes, neutrophils, and platelets, and as elevation of C-reactive protein, adhesion molecules, and proinflammatory cytokines in serum (for review see 1). Previously we have found that platelet aggregates and neutrophils adhere to the vessel lumen after SAH and correlate with local vascular damage [10–12]. In the present investigation we found more intravascular neutrophils and platelet aggregates in SAH males than in females. Gender-related differences in the number of circulating platelets, the degree of platelet adherence to injured vasculature, in agonist-induced platelet activation, and in platelet aggregation have been reported [24,37]. Whereas platelets from females show greater activation and response to adenosine diphosphate or serotonin [38], platelets from men are more responsive to the potent platelet activator, TXA_2_, and less responsive to ADP and serotonin. Moreover, women have higher levels of circulating NO than men [39], a difference which could account for reduced platelet activation and neutrophil activation and reduced vascular adhesion [40] as observed in the present study. Platelets and neutrophils contain collagenases capable of digesting the basal lamina of parenchymal vessels [10,12], and the greater loss of collagen-IV positive vascular basal lamina which we observed in males may be the product of the greater degree of inflammation in those animals. 

Cell death begins early after SAH [16]. Most cell death studies however, have been conducted in male animals, and gender differences in cell death following SAH have not been addressed. By contrast, the effect of gender on cell death following ischemic stroke is well established. Our study finds that apoptotic cell death as revealed by caspase-3 activity and TUNEL staining is greater in males than in females. Fluoro-Jade B staining however did not reveal the same pattern. We think that this result reflects the fact that Fluoro-Jade B stains quiescent and reactive astrocyte and glia and amyloid deposits in brain [41,42]; the greater Fluoro-Jade B staining we observed in females apparently does not reflect greater cell death but rather reports a second gender-sensitive phenomenon of whose nature we are currently ignorant. 

### Gender and 24 hour survival

Our study shows that female rats are more likely to survive the early hours after SAH than their male counterparts. Clinical studies have found no cross-gender differences in outcome after SAH; however, most reports deal with SAH survivors 3-12 months post aneurysm rupture and do not take early (24 hour) deaths in account. The early deaths observed in this study likely correspond to the sudden or early death category which claims almost 43% of SAH victims annually. The few clinical studies which address this question do find suggestions of more frequent early death in men than in women [7–9]. Further study of this question is important.

### Sex hormones and SAH

Our study demonstrates significantly greater early brain injury and significantly reduced survival in male as compared to female rats after SAH. A number of factors may have contributed to the observed poorer outcome in males, including greater hematoma size and greater ICP rise, greater CPP reduction, and greater vascular and inflammatory responses. Moreover, as the animals used in this study were young (6 months old), sex hormones may also have influenced the results [43]. Estrogen decreases the tone of cerebral vessels and increases cerebral blood flow by increasing endothelial production of nitric oxide and prostacyclin [44]. It is well established that PGI2 and NO inhibit platelet activation and adherence to the endothelial wall and reduce inflammation [45]. Estrogen in addition, suppresses apoptosis mechanisms and enhances endothelial cell survival [44]. Consequently, the robust hemostatis, reduced arterial constriction, platelet and neutrophil vascular adherence and apoptosis found in SAH females in the present study may represent estrogen mediated protection. Testosterone, by, enhances the tone of cerebral arteries by suppressing endothelium-dependent vasodilation and by enhancing thromboxane A2 (TxA2) pathway activity [46]. The role of testosterone in SAH pathology has been explored less, but in ischemic stroke testosterone depletion is found to attenuate reperfusion injury, and lesion size [47]. Clinically, although majority of female SAH victims are post menopausal and estrogen loss in women is attributed to the pathogenesis and rupturing of cerebral aneurysm, the importance of sex hormones and outcome is not clear [48,49]. A contribution of sex hormones to the cross-gender difference in intensity of brain injury in our study remains to be studied. 

### Gender, Subarachnoid hemorrhage Hematoma Volume and Brain injury

Similar arterial perforation in the present study resulted in greater bleed volumes in males, as indicated by their greater ICP peak values, as compared to females. Since the volume of bleed is proportionate to the degree of injury and outcome [50], the larger bleeds in the males in this study likely contributed to the greater injury and low survival males experienced. Why males bled more is not clear. Our data do indicate that body size does not correlate with ICP peak or hemorrhage size. Since body blood volume is linearly related to body weight, this indicates that the larger peripheral blood volume in males is not a determining factor for gender differences in the size of hemorrhage. 

We studied correlations between hematoma size and ICP peak with the number of cells dying via apoptosis, and with inflammatory platelets and neutrophils. We found a significant correlation between ICP peak and apoptosis but no correlation between ICP and platelets and neutrophils. This suggests that there may be two separate mechanisms of regulation in play, one that is related to the bleed volume and ICP peak and is directed at apoptosis and a second not directly related to ICP which determines the extent of inflammation. Differences in hematoma size and ICP peak may account for gender differences in apoptotic cell death, but do not account for the observed gender difference in inflammation.

### Translation of Experimental Results to The Clinic

The findings of in this study were made in rat, and translation to human requires further study. As mentioned above, the clinical outcome of SAH in women is not different from than in SAH men [51]. Moreover, a difference in clinical condition on admission (comatose, and clot thickness) is also not observed [3,52]. Gender differences in small vessel pathology and neuronal death reported here in rats have not been studied in SAH patients. There are several reasons for this: 1. injury is of sudden nature; 2. it takes time (as much as 24 hour) for SAH patients to reach a health facility and be diagnosed; 3. a technology that could examine small cerebral vessels in patients for obstruction, inflammation and injury does not exist; and finally 4. the number of early death SAH human brains examined for pathology is small and studies which find microclots and cell death are insufficiently powered to study gender differences. It was for these reasons that we performed this study in the experimental model. Our results establish the importance of pursuing this area in SAH patients

#### Conclusion

We have found that in rats gender influences the initial impact of SAH. Male rats experience a greater bleed, greater severity of early brain injury, and reduced survival as compared to age-matched female rats. 
